# Surgery for Malignant Brain Gliomas: Fluorescence-Guided Resection or Functional-Based Resection?

**DOI:** 10.3389/fsurg.2019.00021

**Published:** 2019-04-12

**Authors:** Hugues Duffau

**Affiliations:** ^1^Department of Neurosurgery, Montpellier University Medical Center, Gui de Chauliac Hospital, Montpellier, France; ^2^Team “Plasticity of Central Nervous System, Stem Cells and Glial Tumors, ” U1051 Laboratory, National Institute for Health and Medical Research (INSERM), Institute for Neurosciences of Montpellier, Montpellier University Medical Center, Montpellier, France

**Keywords:** fluorescence imaging, glioblastoma, functional-based resection, fluorescence-guided resection, brain glioma

The goal of surgery for malignant brain glioma is to optimize the extent of resection (EOR) while preserving the quality of life. A meta-analysis evidenced that gross total resection improves progression free survival and overall survival (OS) in glioblastomas (1). In a consecutive cohort with 500 newly diagnosed glioblastomas, a significant survival benefit was noted with as little as 78% EOR, and stepwise improvement in OS was observed even in the 95–100% EOR range (2). Interestingly, in 243 glioblastomas, survival advantages from total resection remained significant in multivariate analysis after adjustment for bias (3). Concerning anaplastic gliomas, the volume of residual neoplasm on postoperative MRI predicts the time to tumor progression and OS (4).

However, maximal resection can be challenging because it may be difficult to identify the boundaries of glioma due to its infiltrative feature, especially with a white-light microscope. Therefore, to improve the intraoperative real-time visualization of malignant gliomas, the use of fluorescence-guided surgery (FGS) has been advocated, with 5-aminolevulinic acid (5-ALA) as the first option (5, 6). Intrasurgical fluorescence imaging allowed an increase of EOR in high-grade gliomas compared to conventional microsurgery with white-light, as demonstrated by a seminal randomized controlled trial (7). The great merit of this study was also to emphasize the need to objectively assess the EOR on postoperative MRI following glioblastoma surgery. In this Frontiers issue, applications of fluorescence and other optical imaging technology in oncological surgery have been highlighted, especially for malignant brain tumor. Nonetheless, despite a prolific literature on this topic in the past decade, the actual benefit of FGS for glioma patients may be discussed, due to substantial limitations.

From an oncological perspective, beyond the fact that 5-ALA is not adapted to show diffused low-grade gliomas, FGS may paradoxically restrict EOR in high-grade gliomas. Indeed, it has recently been suggested that supracomplete resection, which consists on the removal of a margin around the enhancement, may improve OS in glioblastomas (8). This original concept is based upon the fact that relapses mostly occur at the periphery of the operating cavity, where specific tumor and stromal cells that promote glioblastoma growth and invasion exist (9). In a cohort with 1229 glioblastomas, Li et al. reported a significantly longer OS of 20.7 months when a resection of ≥ 53.21% of the surrounding FLAIR-weighted MRI abnormalities was performed in addition to the total contrast-enhancing removal, versus 15.5 months in the case of excision of the enhancement alone (10). Consequently, even though 5-ALA goes beyond the borders of contrast enhancement, because its diffusion is nonetheless reduced, the tumorological risk intrinsically related to this method is to prematurely stop the resection around the tumor mass identified by fluorescence—while optionally, a lobectomy with a (sub)total resection of the FLAIR abnormalities would have been possible in non-eloquent areas, thus with a better impact on OS. From a functional point of the view, the same property of 5-ALA going beyond the enhanced part of the glioma can result in permanent neurological deterioration for tumors involving structures essential for brain functions. For example, Díez Valle et al. reported a rate of new or increased neurological worsening of 8.2% in a series with glioblastomas operated on using 5-ALA (11), that is, a higher rate in comparison with series using intraoperative electrical mapping −3.4% in a recent meta-analysis (12).

Therefore, an alternative to overcome these limitations is to switch from a FGS to a functional-guided resection by means of direct electrical stimulation (DES) (13). Indeed, the meta-analysis by De Witt et al. in which the benefit of intraoperative electrical mapping on glioma surgery outcome was investigated on the basis of over 8,000 patients, evidenced that the surgical excision of both high-grade gliomas and low-grade gliomas using DES was correlated with more radical resections and with a significantly lower rate of severe permanent impairment—even for tumors located in eloquent regions (12). It is necessary to stress that such a demonstration of an improved EOR associated with a simultaneous decrease of neurological morbidity thanks to the use of fluorescence *per se*, compared with results reported in series using intraoperative functional mapping [currently considered as the standard of care of glioma surgery (12)], is still lacking.

Of note, it has been proposed to combine 5-ALA and electrophysiological mapping, especially for gliomas invading critical areas (14–17). However, even if technically there is not antagonism to use both methods, FGS is conceptually incompatible with functional mapping-based resection. Indeed, although the aim of 5ALA-guided surgery is to remove the enhanced part of the glioma, with the double risk not to achieve a supramarginal resection when functionally feasible or to induce a persistent deficit in eloquent structures (since it is in essence unable to provide functional information), the purpose of mapping-guided surgery is not to achieve a ≪tumorectomy≫ but to perform the most extensive resection of the parenchyma invaded by a diffuse tumoral disease—on the condition that this part of the brain is not critical for neural functions (8, 18). In other words, the aim is to push the resection until eloquent structures have been encountered, both at cortical and subcortical levels, with no margin left around these functional boundaries (13). In practice, if there are discrepancies in information given by 5-ALA and DES, neurosurgeons should rely on functional mapping. For instance, if fluorescence reveals residual glioma but electrical mapping shows that it invades functional tissue, resection must be stopped to preserve the neural networks (19). On the other hand, if 5-ALA demonstrates a ≪complete≫ tumoral removal, but the eloquent structures have not yet been reached according to DES, resection should be pursued up to functional limits in order to achieve a supratotal excision (20) (Figure 1).

**Figure 1:**
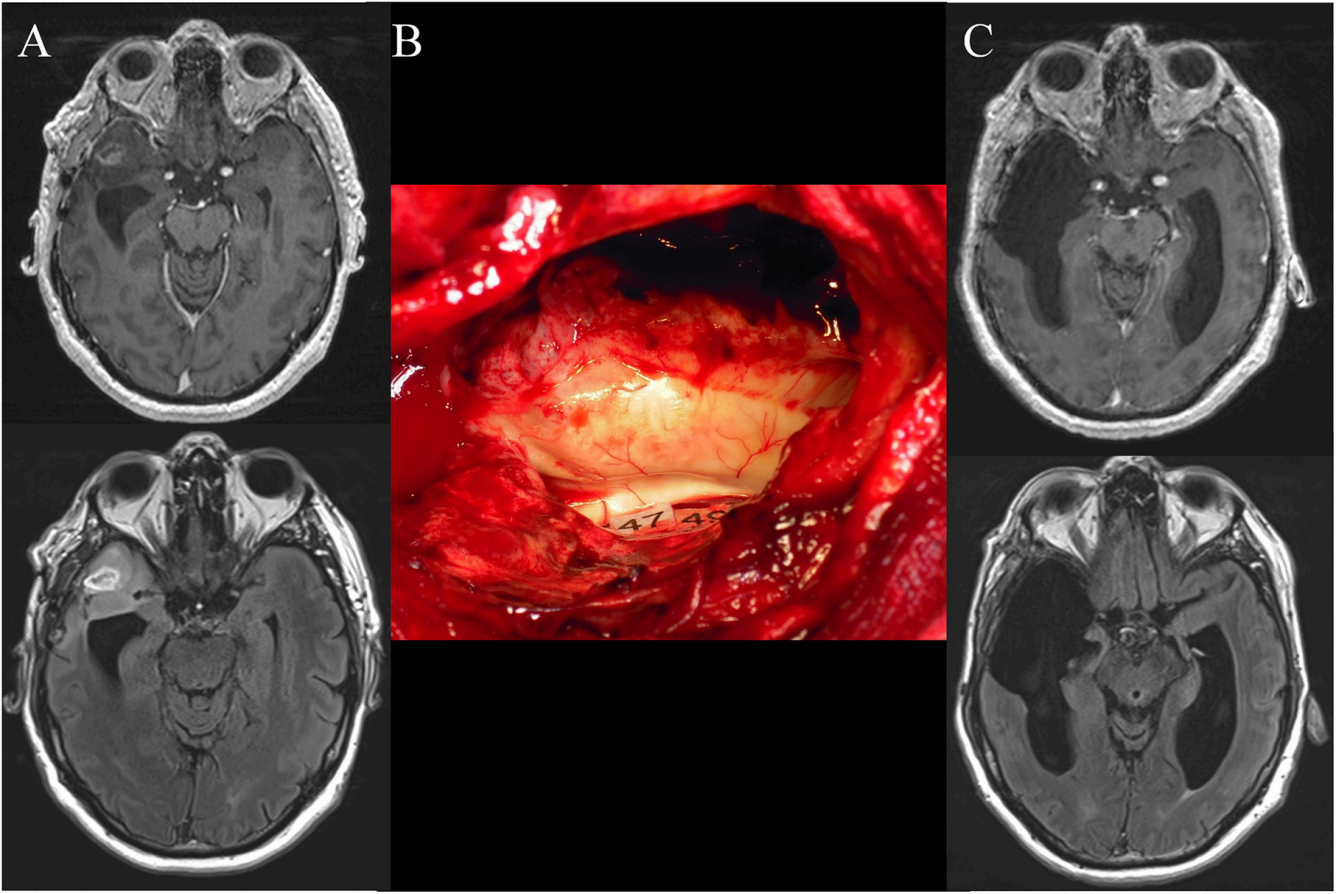
**(A)** Preoperative axial enhanced T1-weighted MRI (upper) and FLAIR-weighted MRI (lower) achieved in a 50-year-old right-handed man who experienced seizures that allowed the discovery of a right anterior temporal high-grade glioma. The patient underwent a previous “minimal-invasive image-guided surgery” performed under general anesthesia in another hospital with a partial resection of the enhancement and the FLAIR hypersignal. An anaplastic astrocytoma was diagnosed. At that time, the patient was referred to our department and a reoperation was proposed with awake mapping in order to achieve a supratotal resection according to functional boundaries. The neurological examination was normal. Nonetheless, the preoperative neuropsychological evaluation revealed a slight deficit of higher-cognitive functions, that is, theory of mind and semantic processing. **(B)** Intraoperative view after resection, achieved up to eloquent structures, especially at the subcortical level. Indeed, direct electrostimulation of white matter tracts enabled the identification of the subcortical neural networks involved in theory of mind (mentalizing) (tag 47) and non-verbal semantics (tag 49) - which have been mapped according to the results of the presurgical neurocognitive assessment. **(C)** Postoperative axial enhanced T1-weighted MRI (upper) and FLAIR-weighted MRI (lower) (performed 3 months after resection) demonstrating a supracomplete resection of both the enhancement and the FLAIR hypersignal. The patient recovered, with an improvement of the neuropsychological examination thanks to a post-surgical cognitive rehabilitation. A diffuse WHO grade III astrocytoma (IDH1 mutated, non-codeleted) was diagnosed, and postoperative chemotherapy was administrated, with no radiotherapy. The imaging is stable with 4 years of follow-up, and the patient continues to enjoy a normal life, with no symptoms.

In summary, with the ultimate goal of optimizing the onco-functional balance, namely, to improve both OS and quality of life in patients with malignant brain gliomas, FGS can be questioned by (re)opening the door to functional mapping-guided resection, to be able to maximize the benefit/risk ratio of surgery in high-grade gliomas (12, 18, 21)—as already extensively demonstrated in diffuse low-grade gliomas (22).

## Author Contributions

The author confirms being the sole contributor of this work and has approved it for publication.

### Conflict of Interest Statement

The author declares that the research was conducted in the absence of any commercial or financial relationships that could be construed as a potential conflict of interest.
